# Unproctored online exams provide meaningful assessment of student learning

**DOI:** 10.1073/pnas.2302020120

**Published:** 2023-07-24

**Authors:** Jason C. K. Chan, Dahwi Ahn

**Affiliations:** ^a^Department of Psychology, Iowa State University, Ames, IA 50014

**Keywords:** online learning, proctoring, assessment, cheating, COVID-19

## Abstract

In the United States, the onset of COVID-19 triggered a nationwide lockdown, which forced many universities to move their primary assessments from invigilated in-person exams to unproctored online exams. This abrupt change occurred midway through the Spring 2020 semester, providing an unprecedented opportunity to investigate whether online exams can provide meaningful assessments of learning relative to in-person exams on a per-student basis. Here, we present data from nearly 2,000 students across 18 courses at a large Midwestern University. Using a meta-analytic approach in which we treated each course as a separate study, we showed that online exams produced scores that highly resembled those from in-person exams at an individual level despite the online exams being unproctored—as demonstrated by a robust correlation between online and in-person exam scores. Moreover, our data showed that cheating was either not widespread or ineffective at boosting scores, and the strong assessment value of online exams was observed regardless of the type of questions asked on the exam, the course level, academic discipline, or class size. We conclude that online exams, even when unproctored, are a viable assessment tool.

## Significance

COVID-19 has vastly expanded the online delivery of higher education. A key question is whether unproctored online exams can accurately assess student learning. To answer this question, we analyzed data from nearly 2,000 students across a wide variety of courses in the Spring 2020 semester, during which the same students had taken both invigilated in-person exams and unproctored online exams. We found that the scores that students obtained during the online exams were highly correlated with their in-person exams. This finding shows that online exams, even when unproctored, can provide a valid and reliable assessment of learning.

The transition of higher learning to online classes has been ongoing since high-speed internet access became widely available (1). COVID-19, however, dramatically accelerated this transition. Online classes make distance education affordable and accessible to millions of students, but a key concern is whether scores from online exams are meaningful.

Success and failure on exams can have a profound impact on a student’s life. For example, failing a required course may delay graduation and impart severe financial distress on the student. Grade-point average is an important determinant of graduate admission outcomes, which can affect a student’s career path. Given the stakes involved, it is not surprising that some students would attempt to achieve better exam scores by cheating, and because online exams are typically taken unmonitored, they provide ample opportunities for dishonest academic behaviors to occur, potentially rendering online exam scores uninformative as a form of assessment (2–4). Echoing this concern, a recent study of 236 faculty in Turkey showed that only 18 respondents expected cheating to be a significant problem in face-to-face education, but 171 indicated the same for online education (5).

As a sign that online exams, even when proctored, are considered inferior to in-person exams, the Medical College Admission Test (MCAT) was never offered online even during the height of the COVID-19 pandemic. In defense of this decision, the Association of American Medical Colleges stated that “there is no way to ensure equal access and test integrity by administering an online proctored exam” (6). Further, in response to the decision to return bar exams to in-person administration in 2021, the National Conference of Bar Examiners claimed that “remote exams create challenges for exam security and uniformity, and… we have consistently advocated for in-person testing as the best option whenever possible” (7).

When researchers compared the scores that students obtain from online unproctored exams against online proctored or in-person exams, the results were somewhat equivocal. Some studies have shown no difference (8–13), whereas others have shown that students score higher on unproctored online exams than either exams given in-person (14, 15) or online with proctoring (16–18), and the unproctored online exam “advantage” varied from just a few percentages (14, 15, 18–20) to nearly 20% (16, 21, 22). Moreover, students tend to spend longer to complete online exams when they are not proctored (17, 20). Together, the higher scores and longer time spent on unproctored online exams have sometimes been interpreted as signs of academic dishonesty. Indeed, survey studies have shown that both faculty and students believe that test takers would be more likely to cheat when taking online exams than in-person exams (23–30). Consequently, some researchers have suggested that, when possible, online exams should be proctored (16, 17).

But there are myriad reasons for online proctoring to reduce test performance outside of its efficacy in preventing cheating. For example, taking an exam while being surveilled can be distracting and anxiety-provoking (8). These negative qualities of online proctoring might suppress students’ test scores. Further, students spending longer on online exams when unproctored does not necessarily indicate cheating (e.g., finding answers in an illicit manner); rather, test takers might simply be more likely to take breaks or to allow household intrusions to occur during an unproctored exam (22, 31, 32). Aside from the controversies surrounding online proctoring and its associated, perhaps prohibitive, financial cost to colleges and universities (33–37), existing data have not provided decisive evidence that cheating is a problem that seriously compromises online exams as a form of assessment at a broad level (38, 39). Our focus is thus on “online exams without proctoring,” which we simply term “online exams” hereafter.

## Online Exams as a Learning Assessment

The critical question is whether scores from unproctored online exams provide meaningful evaluative information about student learning. In the present context, we define a meaningful exam as one that can accurately distinguish students who have learned the materials well from those who have not, such that higher scores on an exam reflect greater learning. Critically, we believe that the relevant comparison is not whether online and in-person exams produce similar scores on average, despite this being the focus of the vast majority of extant studies (2, 8, 9, 12, 14–16, 19, 22, 40), because instructors have many tools to combat score inflation should one exists for online exams relative to in-person exams. For example, instructors can alter grade distributions by changing the grade cutoffs, increase the exam’s difficulty by including more or harder distractors on multiple-choice questions, and ask more application questions rather than definition questions.

We believe that the critical question is whether online and in-person exams provide a similar evaluation of learning—that is, would students who perform well on in-person exams also perform well on online exams? An analogous point was made by the American Psychological Association (APA). Nearly four decades ago (1986), in its report about using computers instead of papers for psychological testing, the APA concluded that “the rank order of individuals’ scores in different modes must approximate each other” for the two forms of assessments to be considered equivalent (41). Likewise, we argue that it is more informative to examine the similarity in how students perform on online and in-person exams at an individual level (i.e., correlation) rather than at a group level (e.g., comparing means).

To investigate whether online and in-person exams produce comparable assessments, the same students must take both online and in-person exams. However, because most studies that examined the comparability of online and in-person exams focused on whether they produce similar scores on average, few have used a within-subjects design that permitted an examination of the assessment value (which we define as the extent to which an exam can accurately determine the degree of learning) for online and in-person exams.^*^ In one study (42), students in an Introductory Psychology hybrid course completed two online quizzes each week and an in-person exam every 3 to 4 wk. The authors found a sizable correlation (*r* = 0.43) between scores on the unproctored online quizzes and the in-person exams (see also 43). This result is promising, but the correlation might be driven, at least partly, by the fact that the online and in-person exams covered the same course content. Specifically, all questions in the quizzes and exams were taken from the textbook’s test bank, with half of the questions used for online quizzes and the remaining used for in-person exams, and each in-person exam covered the topic already tested during the previous two online quizzes.

It is challenging to conduct experimental studies that compare online to in-person exams while maintaining external validity (44), because students in laboratory-based studies do not have the incentives to perform well, so they are unlikely to cheat or to devote the same effort in a laboratory-based study as they do in actual exams (45). In contrast, naturalistic studies typically lack the experimental controls that permit strong conclusions (18), and existing studies almost always featured students in a single course (e.g., Introductory Psychology), thereby limiting their generalizability (15, 40).

However, because of the COVID-19 pandemic, the Spring semester of 2020 (S2020 hereafter) was a singular event that provided the extraordinary conditions to study the comparability of in-person and online exams at a broad level. At many universities, students were taking in-person classes during the first half of the S2020 semester, and those same students, with the same faculty, switched to online classes during the second half of the semester due to the nationwide stay-at-home order in the United States. Because this COVID-induced migration to online learning occurred in March 2020, it conveniently split the semester into two halves. As a result, many instructors had given in-person exams before and online exams after the onset of the COVID lockdown, thus enabling a comparison between these exams on a within-subjects basis. This migration to online learning and assessment also allowed us to conduct a university-wide investigation, a virtually impossible task under any other circumstances.

### Study Setting.

We obtained data from 18 courses offered during S2020 (*N* = 2,010) in a large public university in the Midwestern United States. For the analysis, we calculated an average score for the in-person exams (i.e., first half of the semester) and an average score for the online exams (i.e., second half of the semester) for each student. We then computed the correlation between in-person and online exam scores in each course. We used a meta-analytic approach and treated each course as an individual study, which allowed us to produce an estimate of the overall effect size while taking into account each course’s error variance. This approach also allowed us to examine what might moderate the correlation between online and in-person exam scores. If online exams do not provide a meaningful assessment of learning, then one should expect the correlation between in-person and online exams to be weak to nonexistent. In contrast, assuming that in-person exam is the standard in assessment—and indeed there are currently no practical alternatives—a higher correlation between online and in-person exams indicates greater assessment value for online exams. One might question our assertion that in-person proctored exams provide the best available assessment of student learning, and that many variables can influence the assessment value of an exam. Indeed, exams are by no means perfect, and instructors should use a variety of methods to assess learning (e.g., group discussions, assignments, presentations). But ultimately, every form of assessment has its shortcomings (46), and the society at large has treated in-person exams as the best form of assessment, as evidenced by its use as a gatekeeper for some of the most important or selective professions (47, 48). Moreover, to assess whether educationally relevant variables would affect the assessment value of online exams (relative to in-person exams), we conducted a number of moderator analyses. If the validity of online exam scores is situationally dependent, the correlation between online and in-person exam scores should vary by situational factors such as question type and field of study.

For the sake of completeness, we also examined whether students perform better on online exams than that on in-person exams at the group level. The results of these analyses are presented in Supporting Information.

Because the online exams occurred during the second half of the S2020 semester, they necessarily covered different course content than the in-person exams. Consequently, any discrepancies in performance between online and in-person testing (and an attenuation of the observable correlation) might also be attributed to differences in the content covered across the two halves of the semesters, or that students had become better acquainted with the instructors and the course material during the second half of the semester. To address this issue, we repeated the same analyses using data from instructors who taught the same courses during fully in-person semesters neighboring S2020. We were able to obtain such data for nine courses during 2018, 2019, and 2021 (*N* = 1,072). These data indicate the maximum correlations that one can expect when comparing data from the first and second halves of a semester without changes to exam administration method. Our analyses were preregistered (unless otherwise noted) on the Open Science Framework (OSF) at https://osf.io/3ta9s/?view_only=30843d9b2823473398403ab2dcf91698 (49).

## Results

### Online Exam Scores Are Meaningful.

**Fig. 1.**
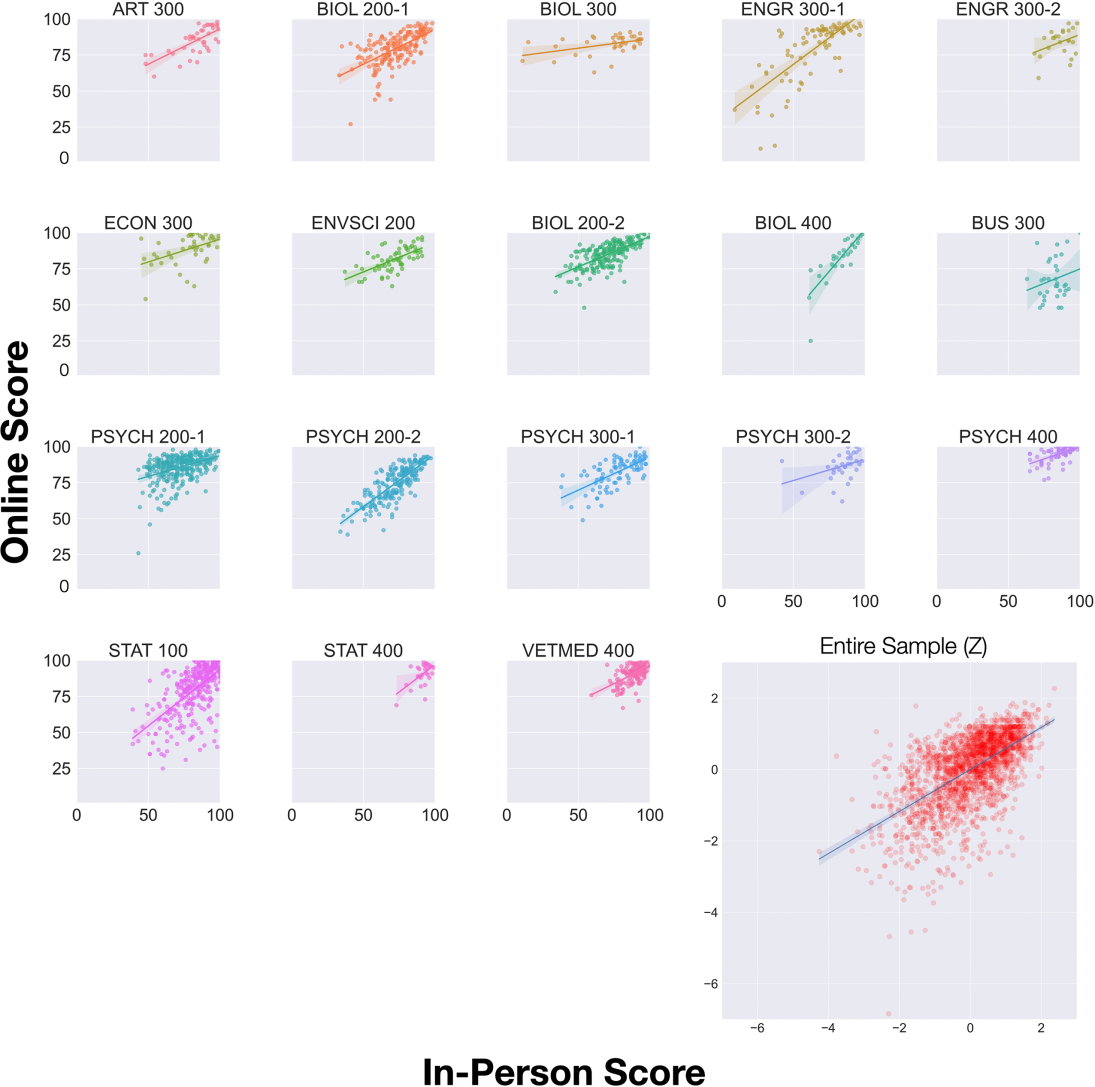
A series of scatterplots of in-person and online exam scores from the S2020 semester. Each small plot shows the data of a single course. The bottom right, larger plot shows data from the entire sample, with the raw scores transformed into coursewise standardized scores. Regression lines were plotted for each course. Each dot shows the data of a single student, with darker color indicating greater data density. As can be seen, every regression line shows a positive association between in-person and online exam scores.

We conducted all analyses under the random effects model. The meta-analytic correlation between online and in-person exams was strongly positive (*r* = 0.59, see Fig. 1). Despite substantial heterogeneity in the data, *Q* = 89.16, *I*^2^ = 81%, a positive correlation was observed for every course, even those with small enrollments. Moderator analyses showed that the correlation between in-person and online exam scores did not vary significantly by types of questions asked on the exams, the field of study, the course level, exam duration, and enrollment. We also investigated whether score inflation for online exams relative to in-person exams (defined as the difference score between online and in-person exams measured in Hedge’s *g*), if one existed, reduced the in-person/online exam score correlation in a meta-regression—it did not. All of the moderator results are shown in Table 1. In sum, scores from the unproctored online exams closely resembled those from the invigilated in-person exams, and this correlation was robust against a host of factors relevant to exam design and administration.

**Table 1.** Influence of moderators on the correlations between in-person and online exam scores

Moderator	Point estimate	0.95 CI	*Q*	*P*	*k*
Question type			0.26	0.612	16
Multiple-choice	0.60	[0.49, 0.70]			9
Open-ended	0.64	[0.49, 0.75]			7
Field of study			0.62	0.432	18
Social sciences, statistics, and humanities	0.57	[0.43, 0.68]			10
Physical sciences and engineering	0.62	[0.51, 0.72]			8
Course level			0.85	0.357	18
Introductory	0.63	[0.48, 0.75]			6
Advanced	0.57	[0.45, 0.67]			12
Exam duration	0.00	[-0.00, 0.01]		0.892	17
Enrollment	0.00	[-0.00, 0.00]		0.639	18
Grade inflation	0.55	[-1.18, 2.30]		0.509	18

**Fig. 2.**
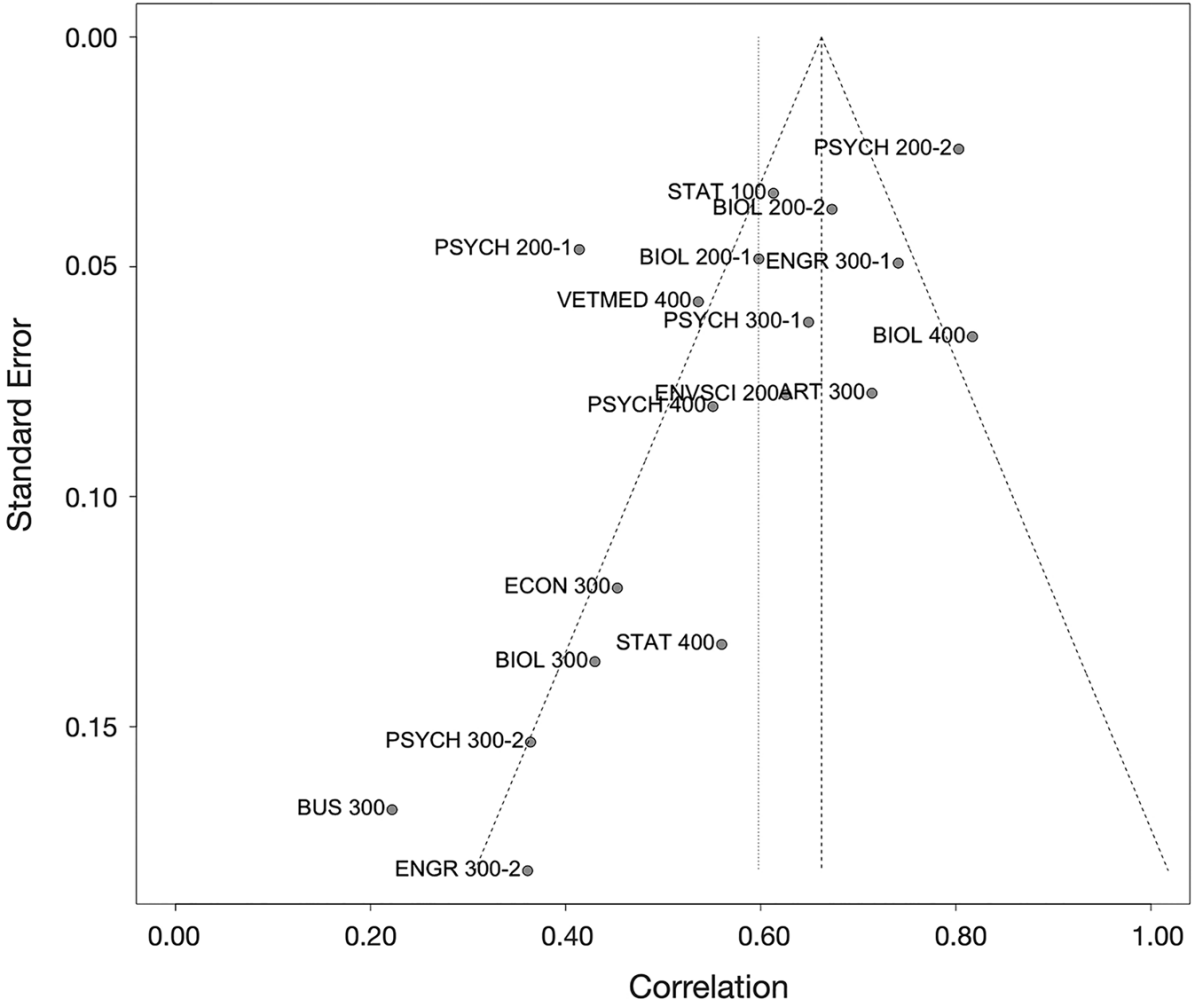
A funnel plot of the correlation effect sizes for data in the S2020 semester. If a study selection bias existed such that we were particularly likely to have recruited instructors who taught small enrollment courses but obtained a strong correlation between their online and in-person exam scores, there should be an overabundance of courses with large error variances to appear on the bottom right of the inverted funnel. The present data exhibited the opposite pattern, which suggests that, if anything, our meta-analytic effect size might be an underestimate of the true effect size.

Although publication bias is not a concern for the present study, we could use analyses meant to address publication bias to detect whether a study selection bias existed in our data. It was possible that our study advertisement disproportionately attracted instructors who taught in ways that produced a strong correlation between in-person and online exam scores, which could occur if instructors who were particularly concerned about the validity of online exams, and who put more effort into ensuring that their online exams were well constructed, were especially likely to respond to our advertisement. Should such a bias existed, one would expect an oversampling of small enrollment (i.e., greater error variance) courses with large effect sizes. The funnel plot in Fig. 2 shows that, if anything, our data exhibited a pattern opposite to such a study selection bias, *Z* = −2.71, *P* = 0.014, with most of the larger error variance studies showing effect sizes on the left side of the funnel. Therefore, we found no evidence that our data recruitment method had led to a selection bias that would inflate our observed effect sizes. If anything, the meta-analytic effect size might have been an underestimate.

Lastly, we examined whether the correlations differed between S2020 and fully in-person semesters. The correlation between scores in the first and second halves of fully in-person semesters was significantly higher than that of Spring 2020 (*r*_other_ = 0.74 vs. *r*_S2020_ = 0.59), *Q* = 5.78, *P* = 0.016. However, when we restricted the analysis to the nine courses taught by the same instructors in both sets of semesters (*k* = 9 each), the difference became smaller and was no longer significant (*r*_other_ = 0.74 vs. *r*_S2020_ = 0.63), *Q* = 2.60, *P* = 0.107. These data suggest that online exams might be inferior to in-person exams in their assessment value, and we address this possibility in the Discussion.

### Is Cheating a Serious Concern for Online Exams?

To assess whether cheating is a serious concern for online exams, we explored the extent to which the association between in-person and online exam scores varied across individuals in a nonpreregistered analysis. The validity of this analysis hinges on three assumptions: First, cheating would increase a student’s score. Second, cheating is less common during invigilated, in-person exams than that during online exams. Third, students’ propensity to cheat during online exams depend on their performance on the in-person exams, such that the worse a student had done on the in-person exams, the more motivated the student would be to cheat during online exams. Existing research provides support for both the second and third assumptions. Regarding the second assumption, it is generally accepted that online exams, even when proctored, offer more opportunities for students to cheat than in-person exams (2, 23–30, 50–52).^†^ Regarding the third assumption, a substantial literature has shown that students with more absences, lower course grades, or lower grade-point average (GPA) are more likely to cheat than their counterparts (53–59). Further, students regularly list performance-related anxieties (e.g., insufficient preparation, exam difficulty, fear of failure) as top reasons to cheat (21, 60–62).

**Fig. 3.**
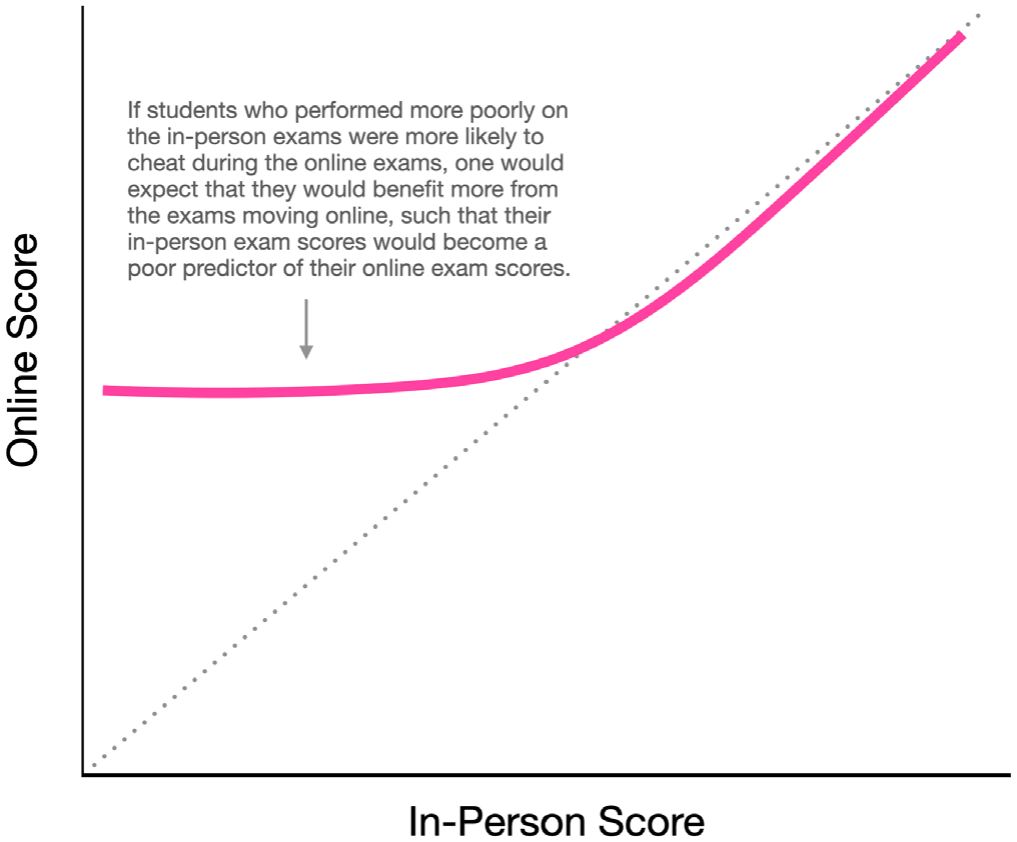
Hypothetical results showing disproportionate cheating during online exams among students who performed poorly on the in-person exams.

The three assumptions described above would lead one to expect a curvilinear relationship between in-person and online exam scores—such that the regression line would be flatter on the left side than that on the right side of the scatterplot, a pattern illustrated in Fig. 3—because students who did poorly on the in-person exams would benefit disproportionately from cheating on the online exams, and their in-person exam scores would become a poor predictor of their online exam scores. For this analysis, we examined the data on an individual basis rather than on a per-class basis using a mega-analysis approach (63, 64). A hierarchical regression showed a robust linear association between in-person and online test scores, *r* = 0.59, *F* = 1,061.58, *P* < 0.001, which was virtually identical to the meta-analytic effect size. More importantly, there was no hint of a nonlinear relationship at all, *r^2^*_change_ = 0.00, *P =* 0.784, *B*_01_ = 27.77. Consequently, our data showed little signs that the online exams were disproportionately advantageous for students who performed poorly on the in-person exams, which suggests that either cheating was not widespread, or perhaps more likely, in contrast to our first assumption for this analysis, cheating was ineffective at boosting exam performance. An additional possibility to consider is that practically everyone cheats during online exams. If this were the case, students who had performed well on the in-person exams should still show less score inflation than those who had performed poorly on the in-person exams (because the former already scored highly on the in-person exams, so they would have less room to inflate their scores by cheating). Our data are consistent with this possibility, but they do not allow us to distinguish whether a larger (but not disproportionately larger) score inflation for low-performing students was due to widespread cheating during online exams, or if it was the consequence of a mathematical necessity (because lower scoring students had more room to improve their scores by cheating than higher scoring students).

## Discussion

Despite the rising importance of unproctored online testing, little research has provided convincing evidence for its value as an assessment tool (38). Here, across a wide variety of courses, we showed that the scores that students obtained from online, unproctored exams resembled those from in-person, proctored exams. A substantial correlation was observed regardless of the level of the course, the content of the course, the type of questions asked on the exams, enrollment, online exam duration, and students’ achievement levels (on the in-person exams). These data showed that, even in highly uncontrolled environments, online exams produced strikingly similar assessments of student learning relative to in-person exams (*r* = 0.59). This effect size is even more impressive when contextualized against the correlation for exam scores between the first and second halves of the semester with only in-person exams (*r* = 0.74), which represents the maximum correlation that one can reasonably expect.

An important implication of these data is that cheating was perhaps uncommon when students took their online exams. Alternatively, if cheating were widespread, then it was not effective at boosting performance. Why might cheating have a minimal effect on performance? We believe that students who feel the need to cheat are likely not performing well in a course. Perhaps they have not attended classes regularly, have not watched the online lectures, have not devoted enough effort to studying, or have not employed effective study strategies, etc. (65). Having access to external materials during a test (e.g., the textbook, notes, or the Internet) does not guarantee good performance, because these students are not familiar with the material. Although this suggestion might seem surprising at first blush, it is consistent with research that showed comparable performance between students who took an exam closed-book or opened-book (11, 66–69), or that students’ performance on opened-book exams can be predicted by their performance on closed-book exams (68, 70, 71).^‡^ To be clear, we are not suggesting that instructors do not need to worry about cheating for online exams at all because organized cheating does occur (72–74), and this type of cheating can seriously jeopardize the validity of all forms of summative or formative assessments. Fortunately, there are effective means to detect and prevent cheating (75, 76). What we have seen from the current dataset, however, is that despite its unproctored nature, online exams produced scores that approximated those from invigilated in-person exams, thereby demonstrating solid validity at a broad level.

A potential concern for the representativeness of our data is that instructors who responded to the advertisement might be especially enthusiastic about teaching and the science of learning. These instructors might be particularly conscientious about online assessments and might put more effort into creating online exams that discourage or minimize cheating (40, 77–80). Consequently, one might interpret the present data as representing the best-case scenario about cheating during online exams. One piece of evidence against this possibility is that our data revealed little signs of a selection bias, so we do not believe that the instructors who contributed data to this study were somehow “different” than those who have not.

### Limitations.

A limitation of the present study was the unique conditions under which students took their online exams. Indeed, what happened during S2020 is unlikely to recur in the foreseeable future. For example, most students probably took their online exams at home during the nationwide lockdown (81). Moreover, students and instructors had to abruptly move to online instructions because of the COVID-19 pandemic. The circumstances under which this migration occurred were unprecedented; therefore, many instructors had little to no practice in teaching and giving exams online (82). Indeed, although we have framed the present study as a comparison between in-person and online exams, the two exam administration methods were also accompanied by a difference in how instructions were delivered (i.e., online exams with online instructions; in-person exams with in-person instructions). In addition, the immense stressors that students felt during the first several months of the COVID-19 pandemic might have decreased the reliability of online exam performance during S2020 relative to more typical circumstances (83–86). When taking these factors into account, the in-person/online correlations observed in the present study might have been an underestimate of the true effect size, so it was perhaps unsurprising that the in-person/online correlations during S2020 were somewhat weaker than those from the non-S2020, fully in-person semesters. At the same time, because most students have not had extensive experience with online courses and exams prior to the S2020 semester, they might be less motivated to cheat during S2020 relative to now (or the future). Indeed, limited recent evidence has suggested that cheating during online exams might be on the rise (35, 87, 88). Assuming that cheating would increase students’ exam scores regardless of their level of learning, an increase in the prevalence of cheating behaviors would reduce the validity of online exam scores. If this were the case, then the strong correlations observed here would be an overestimate of the true effect size going forward.

An advantage of the present dataset is that it contained courses that varied widely in difficulty and students who varied broadly in their academic performance. This heterogeneity was crucial to avoiding the serious issue of range restrictions, which can severely suppress the size of the observed correlations (89). However, despite the broad representation of courses in the current data, they still came from a single public 4-y university in the Midwestern United States, so our conclusions must be considered with this (admittedly common) limitation in mind.

## Conclusions

Using data from the months immediately before and after the onset of the COVID-19 lockdown, we provided a unique dataset that allowed for a comprehensive examination of online exams as an assessment tool. An important assumption of the present research was that we believed in-person exams provide the best assessment of student learning. Although we stand by this argument, we also acknowledge that some factors can affect the validity of in-person exam results. For example, students who suffer from test anxiety might underperform relative to their ability (90). Some students believe that they are “bad test takers,” and this belief might reinforce poor exam preparation behaviors and harm performance (91). Further, some students prefer assignments or group work over exams, which might measure learning beyond that assessed by exams (43).

Our data showed that online exams, even when unproctored, can provide a meaningful assessment of student learning. This encouraging finding, however, does not negate the importance of rethinking assessment for online delivery, especially in light of the explosive growth of generative artificial intelligence tools such as ChatGPT, which can answer complex questions (e.g., essay questions) in ways that are nearly indistinguishable from humans (92–95). We echo recent scholars in advocating for a pivot from memorization-based assessments to ones that emphasize reasoning and application (79). Given that online exams can provide meaningful information about student learning, educators can leverage the powerful advantages of this technology (e.g., instant feedback, easy assignment of a random subset of questions to different students, randomization of choices and question order, easy rescoring of individual questions for an entire class) to deliver better, more authentic assessments.

## Materials and Methods

### Data Collection Practices.

The Institutional Review Board at Iowa State University deemed our study exempt (Institutional Review Board ID 21-209). We collected data from course instructors through advertisements, which included University-wide newsletters distributed by the Center for Excellence in Teaching and Learning (see OSF) and direct emails to all department chairs and the University’s curriculum committee. When instructors responded via email, we asked for anonymized exam data from the S2020 semester. To help standardize data organization, we provided instructors with a sample file containing randomly generated data. We requested instructors to indicate, for each exam, whether it was offered in-person or online, its date, the type of questions asked (e.g., multiple-choice, short-answer questions), its duration, and for how long the exam was available. To ensure that we had a comprehensive dataset, we collected data for nearly 1 y. Specifically, the first advertisement for our study appeared in September 2021 and data collection concluded in August 2022. Table 2 shows the breakdown of courses based on enrollment, major, field, and whether data from non-S2020 semester were provided.

**Table 2.** Details about the courses in the present dataset

Course number	Enrollment	Course name	Major	Other semester data available?
ART 300	41	Art and Architecture of Asia	Art and Art History	No
BIOL 200-1	178	Principles of Biology I	Biology	Yes
BIOL 200-2	214	Introduction to Microbiology	Biology	Yes
BIOL 300	37	Comparative Chordate Anatomy	Biology	No
BIOL 400	27	Bacterial–Plant Interactions	Biology	Yes
BUS 300	33	Database Management Systems	Business	Yes
ECON 300	45	Intermediate Microeconomics	Economics	No
ENGR 300-1	85	Separations Processes	Chemical Engineering	No
ENGR 300-2	24	Electrical Systems in Buildings	Construction Engineering	Yes
ENVSCI 200	62	Biological Processes in the Environment	Environmental Science	Yes
PSYCH 200-1	322	Developmental Psychology	Psychology	No
PSYCH 200-2	213	Social Psychology	Psychology	Yes
PSYCH 300-1	88	Cognitive Psychology	Psychology	Yes
PSYCH 300-2	33	Psychology of Women	Psychology	No
PSYCH 400	76	General Psychopathology	Psychology	No
STAT 100	338	Introduction to Statistics	Statistics	No
STAT 400	28	Regression for Social and Behavioral Research	Statistics	No
VETMED 400	154	Clinical Pathology	Veterinary Pathology	Yes

For comparison purposes, we initially planned to collect data for S2019, but we had to broaden our data collection approach due to logistical reasons (exam data cannot be accessed, the course was not taught by the same instructor or was simply not offered during S2019, etc.). We obtained data from S2018 (*k* = 1), S2019 (*k* = 2), F2019 (*k* = 4), and S2021 (*k* = 2). We classified the exams as belonging to each half of the semester by referring to the data in S2020. For example, if exams 1 to 3 were in-person and exams 4 to 5 were online during S2020 for a given course, then we considered exams 1 to 3 as belonging to the first half and exams 4 to 5 as belonging to the second half for that course in the non-S2020 semesters.

### Missing Data.

If a student missed any exam, the missing exam was not included when calculating the averages. In the S2020 semester, one student missed all of the in-person exams and 11 students missed all of the online exams. We excluded the data from these students in all analyses, such that the final sample contained data from 1,998 students. For data in other semesters, three students missed all of the later half exams, so the final sample contained data from 1,069 students.

### Computational Choices of the Meta-Analysis.

All analyses were done under the random effects model using the *meta* package in *R*. We used the DerSimonian–Laird method to calculate τ2, with Knapp–Hartung adjustments for CIs (96). Correlations were Fisher’s z-transformed for analyses, except for the study selection bias analyses, where using Z would have shown no selection bias.

### Moderators.

#### Question type.

We coded question types into two categories: multiple choice (MC) and open ended (OE). MC included multiple-choice, true-or-false, or matching questions. OE included short answers, essays, calculations, and fill-in-the-blank questions. When both MC and OE questions were used in an exam, the predominant type of questions (i.e., 80% or higher percentage of the score) was used for moderator coding. The course was classified as using open-ended exams if there was no predominant question type. All but two courses (i.e., BUS 300 and ENGR 300-2) employed the same test format for both online and in-person exams. We therefore dropped these two courses from this moderator analysis.

#### Field of study.

We coded this moderator into two categories: Social Sciences, Statistics, & Humanities (SSSH), and Physical Sciences & Engineering (PSE). SSSH included psychology, statistics, economics, business, and art & art history courses. PSE included chemistry, biology, environmental science, veterinary medicine, and engineering courses.

#### Course level.

We coded this variable into an introductory and an advanced level. Introductory included courses at the freshmen and sophomore levels, and advanced included courses at the junior and senior levels.

#### Enrollment.

Enrollment refers to the number of students in a course after missing data were excluded.

#### Grade inflation.

Grade inflation was defined as the difference in average scores for online exams relative to in-person exams. A positive difference indicates inflation, and a negative difference indicates deflation.

#### Exam duration.

Online exam duration was coded in minutes. One course (PSYCH 300-2) gave participants 24 h to complete the exam. We excluded this course from analysis because its duration was outlying (the mean duration for all remaining courses was 81.5 min with a SD of 25 min), but including this course in the analysis did not alter its conclusion.

### Computational Choices of the Mega-Analysis.

We produced two Z scores for each student: one for the in-person exams and one for the online exams. Each Z score was computed by comparing the student’s average (in-person or online) exam score against the mean exam score from the class. Hence, the Z scores represented the student’s performance relative to their classmates rather than to the entire dataset. For the hierarchical regression analysis, we used the online test scores as the dependent variable and the in-person test scores as the predictor in model 1, and we added the squared of the in-person test scores in model 2 to test for a curvilinear relationship.

## Supplementary Material

Appendix 01 (PDF)

## Data Availability

Anonymized behavioral data in csv format have been deposited in OSF (https://doi.org/10.17605/OSF.IO/HMKZG) (49).

## Footnotes

^*^Several studies have examined the correlation between different modes of exams such as open-book vs. closed-book and computer vs. paper (70, 71, 97), but not between online exams and in-person exams or proctored online exams.

^†^Existing research on cheating prevalence has produced wildly different estimates of cheating prevalence (ranging from less than 10% to nearly 100%), although many studies concluded that a majority of college students have cheated (53, 98, 99). Most studies used survey responses to determine cheating prevalence, but they often asked respondents whether they had ever committed any academic dishonesties. These results, therefore, only show that most students had cheated at some point. They do not, however, speak directly to how often or how much students cheat under specific scenarios. For example, a student who answers “yes” to the question “have you ever cheated on a test or written assignment?” could have cheated on a single question during an entire college career. The student could also have cheated regularly, on every question of every exam and assignment. Future research should examine cheating frequency in addition to prevalence. Notably, a similar concern has been voiced in the context of investigating questionable research practices (100).

^‡^Studies that have demonstrated a substantial opened-book superiority typically happened in laboratory settings (101), which do not possess some of the important factors that can affect performance in actual exams (e.g., incentives and stakes, ability to review materials before an exam long after initial learning).

## References

[1] S. A. Ginder, J. E. Kelly-Reid, F. B. Mann, Enrollment and employees in postsecondary institutions, Fall 2017; and financial statistics and academic libraries, fiscal year 2017: first look (provisional data) (NCES 2019-021rev). US Department of Education National Center for Education Statistics (2019). https://nces.ed.gov/pubs2019/2019021REV.pdf.

[2] O. R. Harmon, J. Lambrinos, Are online exams an invitation to cheat? J. Econ. Educ. **39**, 116–125 (2008).

[3] K. Kennedy, S. Nowak, R. Raghuraman, J. Thomas, S. F. Davis, Academic dishonesty and distance learning: Student and faculty views. College Stud. J. **34**, 309–314 (2000).

[4] G. R. Watson, J. Sottile, Cheating in the digital age: Do students cheat more in online courses? *Proceedings of SITE 2008 - Society for Information Technology & Teacher Education International Conference*, 798–803 (2008).

[5] S. Yazici, H. Yildiz Durak, B. Aksu Dünya, B. Şentürk, Online versus face-to-face cheating: The prevalence of cheating behaviours during the pandemic compared to the pre-pandemic among Turkish University students. J. Comput. Assisted Learn. **39**, 231–254 (2023).

[6] American Association of Medical Colleges. The MCAT Exam and COVID-19 (2020). https://www.aamc.org/services/mcat-admissions-officers/faqs-mcat-exam-and-covid-19.

[7] National Conference of Bar Examiners, NCBE anticipates return to in-person testing for February 2022 bar exam (2021). https://www.ncbex.org/news/ncbe-anticipates-return-to-in-person-testing-for-february-2022-bar-exam/.

[8] R. Conijn, A. Kleingeld, U. Matzat, C. Snijders, The fear of big brother: The potential negative side-effects of proctored exams. J. Comput. Assisted Learn. **38**, 1521–1534 (2022).

[9] E. A. Hall, C. Spivey, H. Kendrex, D. E. Havrda, Effects of remote proctoring on composite examination performance among doctor of pharmacy students. Am. J. Pharm. Educ. **85**, 824–828 (2021). 10.5688/ajpe8410 PMC8500287 34615623

[10] K. K. Hollister, M. L. Berenson, Proctored versus unproctored online exams: Studying the impact of exam environment on student performance. Decis. Sci. J. Innov. Educ. **7**, 271–294 (2009).

[11] A. H. Sam, M. D. Reid, A. Amin, High-stakes, remote-access, open-book examinations. Med. Educ. **54**, 767–768 (2020). 32421858 10.1111/medu.14247 PMC7276865

[12] S. Stack, The impact of exam environments on student test scores in online courses. J. Criminal Justice Educ. **26**, 273–282 (2015).

[13] J. R. Stowell, D. Bennett, Effects of online testing on student exam performance and test anxiety. J. Educ. Comput. Res. **42**, 161–171 (2010).

[14] C. Böhmer, N. Feldmann, M. Ibsen, E-exams in engineering education—online testing of engineering competencies: Experiences and lessons learned. 2018 IEEE global engineering education conference (EDUCON) 571–576 (2018), 10.1109/EDUCON.2018.8363281.

[15] M. L. Still, J. D. Still, Contrasting traditional in-class exams with frequent online testing. J. Teach. Learn Tech. **4**, 30–40 (2015).

[16] H. M. Alessio, N. Malay, K. Maurer, A. J. Bailer, B. Rubin, Examining the effect of proctoring on online test scores. Online Learn. **21**, 146–161 (2017).

[17] L. W. Daffin Jr., A. A. Jones, Comparing student performance on proctored and non-proctored exams in online psychology courses. Online Learn. **22,** 131–145 (2018).

[18] B. Means, Y. Toyama, R. Murphy, M. Bakia, K. Jones, Evaluation of evidence-based practices in online learning: A meta-analysis and review of online learning studies. (Department of Education Office of Planning, Evaluation, and Policy Development, Washington DC, US, 2009).

[19] H. Alessio, K. Maurer, The impact of video proctoring in online courses. J. Excel. Col. Teach. **29**, 183–192 (2018).

[20] K. Hylton, Y. Levy, L. P. Dringus, Utilizing webcam-based proctoring to deter misconduct in online exams. Comput. Educ. **92–93**, 53–63 (2016).

[21] H. M. Dodeen, Undergraduate student cheating in exams. Damascus Univ. J. **28**, 37–55 (2012).

[22] P. A. Goedl, G. B. Malla, A study of grade equivalency between proctored and unproctored exams in distance education. Am. J. Dist. Educ. **34**, 280–289 (2020).

[23] S. Aisyah, Y. Bandung, L. B. Subekti, Development of continuous authentication system on android-based online exam application. International conference on information technology systems and innovation (ICITSI) 171–176 (2018).

[24] G. R. Cluskey Jr., C. R. Ehlen, M. H. Raiborn, Thwarting online exam cheating without proctor supervision. J. Acad. Bus Ethics **4**, 1–7 (2011).

[25] O. L. Holden, M. E. Norris, V. A. Kuhlmeier, Academic integrity in online assessment: A research review. Front. Educ. **6** (2021), 10.3389/feduc.2021.639814.

[26] I. Jahnke, J. Liebscher, Three types of integrated course designs for using mobile technologies to support creativity in higher education. Comput. Educ. **146**, 103782 (2020).

[27] P. McGee, Supporting academic honesty in online courses. J. Educ. Online **10**, 1–48 (2013).

[28] F. Noorbehbahani, A. Mohammadi, M. Aminazadeh, A systematic review of research on cheating in online exams from 2010 to 2021. Educ. Inf. Technol. **27**, 8413–8460 (2022). 10.1007/s10639-022-10927-7 PMC8898996 35283658

[29] J. Pagram, M. Cooper, H. Jin, A. Campbell, Tales from the exam room: Trialing an E-exam system for computer education and design and technology students. Educ. Sci. **8**, 188 (2018).

[30] C. F. Rogers, Faculty perceptions about e-cheating during online testing. J. Comput. Sci. Coll. **22**, 206–212 (2006).

[31] K. Hylton, Y. Levy, L. P. Dringus, Utilizing webcam-based proctoring to deter misconduct in online exams. Comput. Educ. **92-93**, 53–63 (2016).

[32] A. Jaap , Effect of remote online exam delivery on student experience and performance in applied knowledge tests. BMC Med. Educ. **21**, 86 (2021). 33530962 10.1186/s12909-021-02521-1 PMC7851803

[33] J. P. Calabrese, Opinion and order on Ogletree v. Cleveland State University. United States District Court Northern District of Ohio Eastern Division (2022).

[34] S. Coghlan, T. Miller, J. Paterson, Good proctor or “Big Brother”? ethics of online exam supervision technologies Philos. Technol. **34**, 1581–1606 (2021). 34485025 10.1007/s13347-021-00476-1 PMC8407138

[35] M. Henderson , Factors associated with online examination cheating. Assess Eval. High Educ. (2022). 10.1080/02602938.2022.2144802.

[36] E. Redden, Rejecting remote proctoring. Inside Higher Ed. (2021). https://www.insidehighered.com/news/2021/04/14/um-dearborn-closed-door-remote-proctoring.

[37] SPARC*, Higher education reckons with concerns over online proctoring and harm to students (2021). https://sparcopen.org/news/2021/higher-education-reckons-with-concerns-over-online-proctoring-and-harm-to-students.

[38] K. Butler-Henderson, J. Crawford, A systematic review of online examinations: A pedagogical innovation for scalable authentication and integrity. Comput. Educ. **159**, 104024 (2020). 32982023 10.1016/j.compedu.2020.104024 PMC7508171

[39] J. A. Rios, O. L. Liu, Online proctored versus unproctored low-stakes internet test administration: Is there differential test-taking behavior and performance. Am. J. Distance Educ. **31**, 226–241 (2017).

[40] B. L. Whisenhunt, C. L. Cathey, D. L. Hudson, L. M. Needy, Maximizing learning while minimizing cheating: New evidence and advice for online multiple-choice exams. Scholarsh Teach Learn Psychol. **8**, 140–153 (2022).

[41] Committee on Professional Standards and Committee on Psychological Tests and Assessment, Guidelines for computer-based tests and interpretations. Am. Psychol Assoc. (1986).

[42] W. S. Maki, R. H. Maki, Mastery quizzes on the web: Results from a web-based introductory psychology course. Behav. Res. Methods, Instr. Comput. **33**, 212–216 (2001). 10.3758/bf03195367 11447674

[43] G. Smith, How does student performance on formative assessments relate to learning assessed by exams? J. Coll. Sci. Teach. **36**, 28–34 (2007).

[44] K. Shankar, P. Arora, M. C. Binz-Scharf, Evidence on online higher education: The promise of COVID-19 pandemic data. Manage. Labour Stud. **48**, 242-249 (2021).

[45] M. N. Karim, S. E. Kaminsky, T. S. Behrend, Cheating, reactions, and performance in remotely proctored testing: An exploratory experimental study. J. Business Psychol. **29**, 555–572 (2014).

[46] S. Shaw, Traditional examinations: the best form of assessment? The Student (2018). https://studentnewspaper.org/traditional-examinations-the-best-form-of-assessment/.

[47] R. C. Rabin, Want to be a doctor? (Take your chances in a closed room with strangers. NY Times, 2020.

[48] D. C. Weiss, Online bar exams axed by NCBE beginning next year (2021). https://www.abajournal.com/news/article/online-bar-exams-axed-by-ncbe-beginning-next-year.

[49] D. Ahn, J. C. K. Chan, Are online exams as effective as in-person exams? - preliminary analysis. OSF Registries. https://osf.io/hmkzg5. Deposited 9 October 2022.

[50] I. J. M. Arnold, Cheating at online formative tests: Does it pay off. Internet and Higher Educ. **29**, 98–106 (2016).

[51] S. Dendir, R. S. Maxwell, Cheating in online courses: Evidence from online proctoring. Comput. Hum. Behav. Rep. **2**, 100033 (2020).

[52] R. N. Tarigan, R. Nadlifatin, A. P. Subriadi, Academic Dishonesty (Cheating) In Online Examination: A Literature Review. 2021 International Conference on Computer Science, Information Technology, and Electrical Engineering (ICOMITEE) (2021). 10.1109/icomitee53461.2021.9650082.

[53] B. E. Whitley, Factors associated with cheating among college students: A review. Res. Higher Educ. **39**, 235–274 (1998).

[54] A. L. Bronzaft, I. R. Stuart, B. Blum, Test anxiety and cheating on college examinations. Psychol. Rep. **32**, 149–150 (1973). 4686059 10.2466/pr0.1973.32.1.149

[55] D. N. Bunn, S. B. Caudill, D. M. Gropper, Crime in the classroom: An economic analysis of undergraduate student cheating behavior. J. Econ. Educ. **23**, 197–207 (1992).

[56] R. T. Burrus, K. McGoldrick, P. W. Schuhmann, Self-reports of student cheating: Does a definition of cheating matter. J. Econ. Educ. **38**, 3–16 (2007).

[57] J. A. Davy, J. F. Kincaid, K. J. Smith, M. A. Trawick, An examination of the role of attitudinal characteristics and motivation on the cheating behavior of business students. Ethics amp Behav. **17**, 281–302 (2007).

[58] G. M. Diekhoff , College cheating: Ten years later. Res. Higher Educ. **37**, 487–502 (1996).

[59] C. Nowell, D. Laufer, Undergraduate student cheating in the fields of business and economics. J. Econ. Educ. **28**, 3–12 (1997).

[60] M. Hosny, S. Fatima, Attitude of students towards cheating and plagiarism: University case study. J. Appl. Sci. **14**, 748–757 (2014).

[61] T. B. Murdock, E. M. Anderman, Motivational perspectives on student cheating: Toward an integrated model of academic dishonesty. Educ. Psychol. **41**, 129–145 (2006).

[62] J. Sheard, S. Markham, M. Dick, Investigating differences in cheating behaviours of IT undergraduate and graduate students: The maturity and motivation factors. Higher Educ. Res. Develop. **22**, 91–108 (2003).

[63] P. J. Curran, A. M. Hussong, Integrative data analysis: the simultaneous analysis of multiple data sets. Psychol. Methods **14**, 81–100 (2009). 19485623 10.1037/a0015914 PMC2777640

[64] J. G. Eisenhauer, Meta-analysis and mega-analysis: A simple introduction. Teaching Stat. **43**, 21–27 (2021).

[65] S. D. Rea, L. Wang, K. Muenks, V. X. Yan, Students can (mostly) recognize effective learning, so why do they not do it. J. Intell. **10**, 127 (2022). 36547514 10.3390/jintelligence10040127 PMC9781761

[66] R. M. Block, A discussion of the effect of open-book and closed-book exams on student achievement in an introductory statistics course. PRIMUS **22**, 228–238 (2012).

[67] S. J. Durning , Comparing open-book and closed-book examinations: A systematic review. Acad. Med. **91**, 583–599 (2016). 26535862 10.1097/ACM.0000000000000977

[68] M. Heijne-penninga, J. B. M. Kuks, J. Schönrock-adema, T. A. B. Snijders, J. Cohen-schotanus, Open-book tests to complement assessment-programmes: Analysis of open and closed-book tests. Adv. Health Sci. Educ. **13**, 263–273 (2008). 10.1007/s10459-006-9038-y 17063381

[69] M. K. Ioannidou, Testing and life-long learning: Open-book and closed-book examination in a university course. Stud. Educ. Eval. **23**, 131–139 (1997).

[70] D. Boniface, Candidates’ use of notes and textbooks during an open-book examination. Educ. Res. **27**, 201–209 (1985).

[71] A. Gharib, W. Phillips, N. Mathew, Cheat sheet or open-book? A comparison of the effects of exam types on performance, retention, and anxiety. Psychol. Res. **2**, 469–478 (2012).

[72] S. E. Eaton, Academic Integrity in Canada: An Enduring and Essential Challenge, S. E. Eaton, J. Christensen Hughes, Eds. (Springer International Publishing, Cham, 2022), pp. 165–188.

[73] R. F. Parks, P. B. Lowry, R. T. Wigand, N. Agarwal, T. L. Williams, Why students engage in cyber-cheating through a collective movement: A case of deviance and collusion. Comput. Educ. **125**, 308–326 (2018).

[74] J. R. Young, Online classes see cheating go high tech. Chronicle of Higher Educ. (2013). https://www.chronicle.com/article/online-classes-see-cheating-go-high-tech.

[75] H. Abood, M. A. Maizer, Strategies to address cheating in online exams. Int. J. Tech. Educ. **5**, 608–620 (2022).

[76] S. Kaddoura, A. Gumaei, Towards effective and efficient online exam systems using deep learning-based cheating detection approach. Intell. Syst. Appl. **16**, 200153 (2022).

[77] A. C. Butler, Multiple-choice testing in education: Are the best practices for assessment also good for learning. J. Appl. Res. Mem. Cogn. **7**, 323–331 (2018).

[78] D. Harrison, Online education and authentic assessment. Inside Higher Ed. (2020). https://www.insidehighered.com/advice/2020/04/29/how-discourage-student-cheating-online-exams-opinion.

[79] M. Schultz, D. L. Callahan, Perils and promise of online exams. Nat. Rev. Chem. **6**, 299–300 (2022). 35402730 10.1038/s41570-022-00385-7 PMC8981880

[80] X. Xu, S. Kauer, S. Tupy, Multiple-choice questions: Tips for optimizing assessment in-seat and online. Scholarsh Teach. Learn Psychol. **2**, 147–158 (2016).

[81] J. Piccirillo, The country is reopening — now what? (2020). https://www.yalemedicine.org/news/covid-country-reopening.

[82] R. B. Moralista, R. M. F. Oducado, Faculty perception toward online education in a state college in the Philippines during the Coronavirus Disease 19 (COVID-19) pandemic. Univ. J. Educ. Res. **8**, 4736–4742 (2020).

[83] A. M. Lederer, M. T. Hoban, S. K. Lipson, S. Zhou, D. Eisenberg, More than inconvenienced: The unique needs of US college students during the COVID-19 pandemic. Health Educ. Behav. **48**, 14–19 (2021). 33131325 10.1177/1090198120969372 PMC8356799

[84] Y. Li, A. Wang, Y. Wu, N. Han, H. Huang, Impact of the COVID-19 pandemic on the mental health of college students: A systematic review and meta-analysis. Front. Psychol. **12**, 669119 (2021). 34335381 10.3389/fpsyg.2021.669119 PMC8316976

[85] X. Wang , Investigating mental health of US college students during the COVID-19 pandemic: Cross-sectional survey study. J. Med. Int. Res. **22**, e22817 (2020). 10.2196/22817 PMC7505693 32897868

[86] C. Wang , Anxiety, depression, and stress prevalence among college students during the COVID-19 pandemic: A systematic review and meta-analysis. J. Am. Coll Health 1–8 (2021), 10.1080/07448481.2021.1960849. 34469261

[87] P. Newton, K. Essex, How common is cheating in online exams and did it increase during the COVID-19 pandemic? A Systematic Review. Preprint (2023). 10.21203/rs.3.rs-2187710/v1 (Accessed 24 January 2023).

[88] T. Williams, Online exam cheating is up. Inside Higher Ed. (2022). https://www.insidehighered.com/news/2022/04/28/study-online-exam-cheating.

[89] D. S. Lindsay, J. D. Read, K. Sharma, Accuracy and confidence in person identification: The relationship is strong when witnessing conditions vary widely. Psychol. Sci. **9**, 215–218 (1998).

[90] S. J. Myers, S. D. Davis, J. C. K. Chan, Does expressive writing or an instructional intervention reduce the impacts of test anxiety in a college classroom. Cogn. Res. Princ. Implic **6**, 44 (2021). 34114117 10.1186/s41235-021-00309-x PMC8192598

[91] K. J. Friston , Event-related fMRI: Characterizing differential responses. Neuroimage **7**, 30–40 (1998). 9500830 10.1006/nimg.1997.0306

[92] J. de Winter , Can ChatGPT pass high school exams on English language comprehension? Researchgate Preprint (2023) (Accessed 25 January 2023).

[93] A. Gilson , Does ChatGPT perform on the medical licensing exams? The implications of large language models for medical education and knowledge assessment. medRxiv [Preprint] (2023). 10.1101/2022.12.23.22283901 (Accessed 26 December 2022). PMC9947764 36753318

[94] K. Huang, Alarmed by A.I. chatbots, universities start revamping how they teach. NY Times, 2023.

[95] T. Susnjak, ChatGPT: The end of online exam integrity. arXiv [Preprint] (2023). 10.48550/arXiv.2212.09292 (Accessed 25 January 2023).

[96] G. Knapp, J. Hartung, Improved tests for a random effects meta-regression with a single covariate. Stat. Med. **22**, 2693–2710 (2003). 12939780 10.1002/sim.1482

[97] I.-C. Choi, K. S. Kim, J. Boo, Comparability of a paper-based language test and a computer-based language test. Lang. Testing **20**, 295–320 (2003).

[98] D. L. McCabe, Cheating among college and university students: A North American perspective. Int. J. Ed. Integr. **1** (2005).

[99] D. L. McCabe, L. K. Treviño, K. D. Butterfield, Cheating in academic institutions: A decade of research. Ethics Behav. **11**, 219–232 (2001).

[100] K. Fiedler, N. Schwarz, Questionable research practices revisited. Soc. Psychol. Personality Sci. **7**, 45–52 (2015).

[101] P. K. Agarwal, J. D. Karpicke, S. H. K. Kang, H. L. Roediger, K. B. McDermott, Examining the testing effect with open- and closed-book tests. Appl. Cogn. Psychol. **22**, 861–876 (2008).

