# Supplementary material for: Unproctored online exams provide meaningful assessment of student learning
Source: Proc Natl Acad Sci U S A. 2023 Jul 24;120(31):e2302020120. doi: 10.1073/pnas.2302020120 (PMC10400982; doi:10.1073/pnas.2302020120)
Supplement: Supplementary file 1 — Appendix 01 (PDF) [file pnas.2302020120.sapp.pdf]

PNAS

Supporting Information for
Unproctored Online Exams Provide Meaningful Assessment of Student Learning

Jason C.K. Chan; Dahwi Ahn

Paste corresponding author name here: Jason C.K. Chan, Dahwi Ahn

Email: ckchan@iastate.edu; dahn@iastate.edu

This PDF file includes:

Supporting text
Figures S1
Tables S1
SI References

Supporting Information Text

Did students score higher on online exams than on in-person exams?

As we have mentioned in the main text of the manuscript, comparing the average scores of online exams and in-person exams at the group level is not particularly informative given that i) the two sets of exams did not cover the same material, and ii) instructors might have created the exam questions with different approaches (e.g., the in-person exams might have featured more fact-based questions, whereas the online exams might have featured more application questions).[1] But we present the results of these analyses here for the sake of completeness. Note that these potential issues, should they exist, would also affect correlations as well, but they would likely attenuate, rather than strengthen, our observed correlations.

For these analyses, we computed the standardized mean difference (Hedge's g) between online and in-person exam scores for each course. We then pooled the effect sizes across courses using a meta-analytic approach.

Consistent with some prior literature, our data showed that students scored modestly higher on online exams than on in-person exams, $g = 0.40$, $t = 2.85$, $p = .011$. The funnel plot in Figure S1 shows that there is no obvious study selection bias, Z = 1.86, $p = .529$. In raw percentage terms, students scored about 5% higher on the online exams than on the in-person exams. Question type moderated the difference in exam scores, $Q = 3.91$, $p = .048$, such that exams that were primarily multiple-choice showed greater grade inflation ($g = 0.70$) than those that featured more open-ended questions ($g = 0.27$). None

[1] One might note that these "issues" also apply when we use correlation as our dependent measure. But in the case of correlations, changes that instructors implement to the online exams would likely *attenuate* the correlation between scores on the in-person and online exams, which would work against us discovering a strong correlation.

of the other variables (i.e., field of study, course level, exam duration, enrollment) significantly moderated the score inflation associated with online exams (see Table S1).

To ascertain that the score inflation observed in the second half of S2020 (relative to the first half) was due to the switch to online exams rather than students generally performing better during the second half of the semester, we also compared students' scores between the first and second half of the fully in-person semesters. Unlike S2020 (g_{S2020} = 0.40), there was no evidence of grade inflation in the second half relative to the first half of the purely in-person semesters (g_{other} = 0.06), t = 0.36, p = .731. However, when we examined the data for the subset of S2020 courses that were taught by the same instructors in both sets of semesters, the S2020 data no longer exhibited significant signs of grade inflation, g = 0.28, t = 1.30, p = .229.

Discussion

Our data showed that students scored higher on the online exams than on the in-person exams. Although this score inflation was not large, it occurred under the unfavorable conditions of the COVID-19 lockdown, so it is possible that the score inflation observed here was an underestimate. However, as we have noted, we do not find average differences between online and in-person exams to be particularly informative, and many methods can combat score inflations that occurred at a group level, including adjusting the grade cutoffs, creating more challenging questions, adding more response options to multiple choice questions, or implementing more recall type (e.g., short-answer, essay questions). For example, our data suggest that exams with more open-ended questions might be less susceptible to the score inflations associated with online exams than exams with mostly multiple-choice questions.

If cheating is not a major concern for online exams, then why would students score higher on them than on in-person exams? There are several possibilities. First, some students find online exams less anxiety-inducing because they can take the exam in a familiar environment and by themselves, or they might feel less time pressure during online exams than in-person exams (1, 2). Second, instructors might be more inclined to deploy multiple-choice than short-answer or essay questions in online exams relative to in-person exams. All else being equal, multiple-choice questions are easier than open-ended questions, so if instructors were more likely to use multiple-choice questions in online than in-person exams, then scores on the online exams can be expected to increase relative to in-person exams.

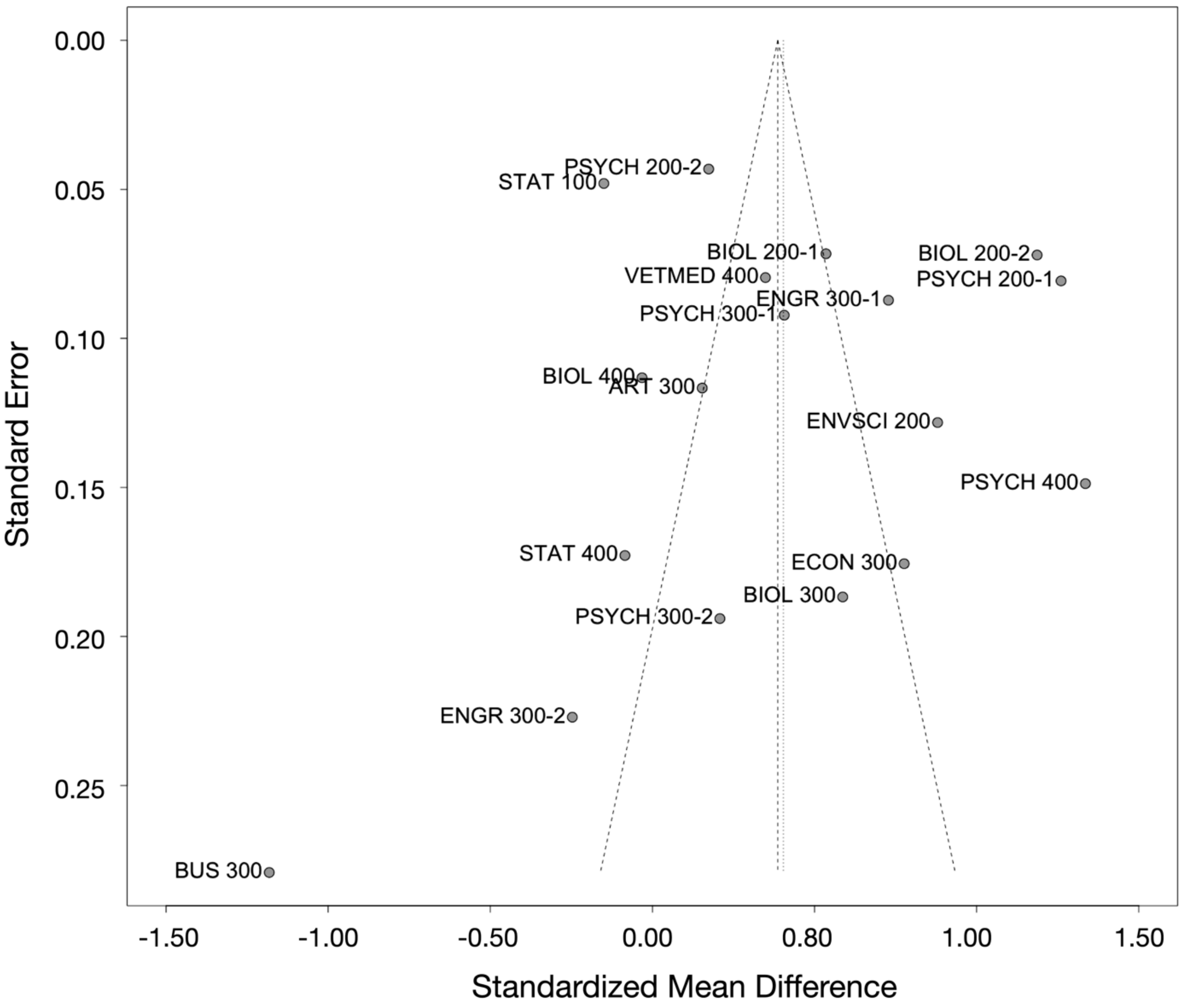

Figure S1.

Funnel Plot of the effect sizes showing the standardized difference in test scores between in-person and online exams for data in the S2020 semester. A positive effect size indicates that students scored higher on the online exams than on the in-person exams for a given class.

Table S1.
Influence of moderators on the mean difference between in-person and online exam scores

Moderator	Point Estimate	.95 Confidence Interval	*Q*	*p*	*k*
Test Format			3.91	.048	16
Multiple-choice	0.70	[0.35, 1.06]			9
Open-ended	0.27	[-0.10, 0.65]			7
Field of Study			0.54	.464	18
Social sciences, statistics, & humanities	0.31	[-0.19, 0.82]			10
Physical sciences & engineering	0.52	[0.13, 0.90]			8
Course Level			1.57	.210	18
Introductory	0.64	[0.05, 1.24]			6
Advanced	0.28	[-0.10, 0.66]			12
Exam Duration	-0.00	[-0.02, 0.01]		.476	17
Enrollment	0.00	[-0.00, 0.00]		.281	18

SI References

1. Stowell JR, Bennett D, Effects of online testing on student exam performance and test anxiety. *J Educ Comput Res* 42(2), 161–171. (2010).
2. Williams JB, Wong A, The efficacy of final examinations: A comparative study of closed-book, invigilated exams and open-book, open-web exams. *Br J Educ Technol* 40(2), 227–236. (2009).
